# A comparison of machine learning techniques for classification of HIV patients with antiretroviral therapy-induced mitochondrial toxicity from those without mitochondrial toxicity

**DOI:** 10.1186/s12874-019-0848-z

**Published:** 2019-11-27

**Authors:** Jong Soo Lee, Elijah Paintsil, Vivek Gopalakrishnan, Musie Ghebremichael

**Affiliations:** 1 Department of Mathematical Sciences, University of Massachusetts, Lowell, MA USA; 2 Department of Pediatrics, Yale University, New Haven, CT USA; 3 Department of Biomedical Engineering, The Johns Hopkins University, Baltimore, MD USA; 4 Ragon Institute of Harvard, MGH and MIT, 400 Technology Square, Cambridge, MA 02129 USA

**Keywords:** HIV/AIDS, Machine learning, Classification, Dimension reduction, Mitochondrial toxicity, Antiretroviral therapy

## Abstract

**Background:**

Antiretroviral therapy (ART) has significantly reduced HIV-related morbidity and mortality. However, therapeutic benefit of ART is often limited by delayed drug-associated toxicity. Nucleoside reverse transcriptase inhibitors (NRTIs) are the backbone of ART regimens. NRTIs compete with endogenous deoxyribonucleotide triphosphates (dNTPs) in incorporation into elongating DNA chain resulting in their cytotoxic or antiviral effect. Thus, the efficacy of NRTIs could be affected by direct competition with endogenous dNTPs and/or feedback inhibition of their metabolic enzymes. In this paper, we assessed whether the levels of ribonucleotides (RN) and dNTP pool sizes can be used as biomarkers in distinguishing between HIV-infected patients with ART-induced mitochondrial toxicity and HIV-infected patients without toxicity.

**Methods:**

We used data collected through a case-control study from 50 subjects. Cases were defined as HIV-infected individuals with clinical and/or laboratory evidence of mitochondrial toxicity. Each case was age, gender, and race matched with an HIV-positive without evidence of toxicity. We used a range of machine learning procedures to distinguish between patients with and without toxicity. Using resampling methods like Monte Carlo *k*-fold cross validation, we compared the accuracy of several machine learning algorithms applied to our data. We used the algorithm with highest classification accuracy rate in evaluating the diagnostic performance of 12 RN and 14 dNTP pool sizes as biomarkers of mitochondrial toxicity.

**Results:**

We used eight classification algorithms to assess the diagnostic performance of RN and dNTP pool sizes distinguishing HIV patients with and without NRTI-associated mitochondrial toxicity. The algorithms resulted in cross-validated classification rates of 0.65–0.76 for dNTP and 0.72–0.83 for RN, following reduction of the dimensionality of the input data. The reduction of input variables improved the classification performance of the algorithms, with the most pronounced improvement for RN. Complex tree-based methods worked the best for both the deoxyribose dataset (Random Forest) and the ribose dataset (Classification Tree and AdaBoost), but it is worth noting that simple methods such as Linear Discriminant Analysis and Logistic Regression were very competitive in terms of classification performance.

**Conclusions:**

Our finding of changes in RN and dNTP pools in participants with mitochondrial toxicity validates the importance of dNTP pools in mitochondrial function. Hence, levels of RN and dNTP pools can be used as biomarkers of ART-induced mitochondrial toxicity.

## Background

Although significant progress has been made to contain the AIDS epidemic, the Joint United Nations Program on HIV/AIDS (UNAIDS) estimates that there are currently over 36.9 [34.3–41.4] million people globally living with HIV [UNAIDS, 2015]. According to UNAIDS, 1.20 [0.98–1.60] million people died from AIDS-related causes worldwide in 2014. Antiretroviral therapy (ART) has significantly reduced HIV-related morbidity and mortality worldwide, however, its therapeutic benefit is compromised by delayed drug-associated toxicity [1]. A recent update from the Italian of Cohort Naïve Antiretrovirals (ICONA) Foundation study showed the 1-year probability of discontinuation of ART due to toxicity was 19% for patients who initiated ART between 2008 and 2014 [2]. Nucleoside reverse transcriptase inhibitors (NRTIs), the first class of ART used in the treatment of individuals with HIV, have been associated with toxicities that mirror symptoms in individuals with mitochondrial disorders. The presentation of NRTI-induced mitochondrial toxicity includes lactic acidosis, lipodystrophy, peripheral neuropathies, cardiomyopathies, and pancytopenia [1, 3–6]. These manifestations have been ascribed to the inhibitory effect of NRTIs on polymerase gamma (Pol-ɣ), the enzyme that replicates mitochondrial DNA (mtDNA). Inhibition of Pol-ɣ leads to depletion of mtDNA content and subsequent mitochondrial dysfunction. However, there are emerging reports that other classes of ART such as nonnucleoside reverse transcriptase inhibitors (NNRTIs) and protease inhibitors (PIs) can cause mitochondrial dysfunction through Pol-ɣ-independent mechanisms [7, 8].

We recently reported that HIV-infected patients on NRTI-based ART with mitochondrial toxicity tended to have decreased concentrations of both ribonucleotide (RN) and deoxyribonucleotide (dNTP) pool sizes (precursors of DNA synthesis) in their peripheral blood mononuclear cells (PBMCs) [9]. Interestingly, patients with mitochondrial toxicity had statistically significant higher mRNA expression of Pol-ɣ compared to patients without mitochondrial toxicity. In this paper, we aimed to determine the effectiveness of RN and dNTP pool sizes as biomarkers in distinguishing between HIV patients with and without mitochondrial toxicity. More specifically, the objective of this paper is two-fold: First, we assessed whether the levels of RN and dNTP pool sizes can be used as biomarkers in distinguishing between HIV-infected patients with ART-induced mitochondrial toxicity and HIV-infected patients without toxicity. Second, we assessed whether a range of machine learning procedures could distinguish between patients with and without toxicity. In this study, where we have both the limited sample size and dataset with varying correlation structure, it will be particularly prudent to consider many different machine learning algorithms to assess their performances in a meaningful way. We used previously acquired data on ART-induced mitochondrial toxicity to evaluate the relative performance of eight different machine learning procedures. Using resampling methods like Monte Carlo *k*-fold cross validation, we compared the accuracy of the machine learning algorithms applied to our data. We ran a large set of simulation studies to evaluate the performance of the machine learning algorithms for varying sample sizes and correlation between RN and dNTP pool sizes. We used the algorithm with highest classification accuracy rate in evaluating the diagnostic performance of RN and dNTP pool sizes as biomarkers of mitochondrial toxicity.

## Methods

### Study participants

The study included 50 HIV-infected individuals on NRTI-based ART regimens enrolled through a case-control study from April 2011 to March 2013. Twenty-five of the HIV-infected individuals were diagnosed by their providers as having mitochondrial toxicity based on at least one clinical or laboratory findings associated with NRTI toxicity (i.e., lactic acidemia, pancreatitis, peripheral neuropathy, lipodystrophy, creatinine, anemia, renal dysfunction, elevated liver enzymes, hyperlipidemia, amylase, and lipase) [1, 3–6]. The study participants were recruited from the Adults AIDS Care Programs at Yale-New Haven Hospital. At enrollment participants answered a brief survey comprised of questions regarding past medical history and demographic characteristics. Medical records of participants were reviewed for date of HIV diagnosis, medication history, date of diagnosis of toxicity, complete blood count, serum chemistries, liver function test, lipid profile, urinalysis, HIV RNA copy number, and CD4+ T-cell count at study entry. Blood was collected from all study participants at the time of enrollment for extraction and quantification of ribonucleotide (RN) and deoxyribonucleotide (dNTP) pool sizes. The study protocol was approved by the Institutional Review Board of the Yale University School of Medicine. Informed and written consents were obtained from study participations prior to enrollment in the study. The rationale, organization, and recruitment of the subjects, biological procedures used for extraction and quantification RN and dNTP pools have been described previously [9].

### Statistical methods

#### Data classification methods

There are many classification methods, some of which can be very sophisticated and state-of-the-art [10]. In this paper, we focused on the most common methods which are readily available and can easily be implemented in most statistical packages. We briefly described the classification methods used in the paper below:

##### A. Linear and quadratic discriminant analysis

The linear discriminant analysis (LDA) and the quadratic discriminant analysis (QDA) are classical statistical classification methods [11]. In classifying two groups, both methods incorporate log likelihood ratio based on normal distribution, with the main difference that LDA assumes the equal covariance while QDA does not.

##### B. K-nearest neighbor

The k-nearest neighbor (KNN) is a simple classification method, using the k nearest points of the input to predict the response [12]. Here, we first predict the value *Y* based on *x*, by

$$ \hat{Y}(x)=\frac{1}{k}\sum \limits_{x_i\in {N}_k(x)}{y}_i, $$

where *N*_*k*_(*x*) is the neighborhood of *x* defined by the *k* closest input points *x*_*i*_ to x (using Euclidean distance in most cases), and k values of *y*_*i*_ corresponding to *x*_*i*_, where *y*_*i*_ = 0 or *y*_*i*_ = 1, at each neighborhood *N*_*k*_(*x*). Hence, $\hat{Y}(x)$ is the proportion of time that *y*_*i*_ = 1 in the neighborhood *N*_*k*_(*x*) for the point x, and we make the final decision as 1 if $\hat{Y}(x)$ > 0.5 and as 0 otherwise, at each point x. We may use different values of *k* for the *k*-neighbor. As *k* decreases, the model can be more accurate on the training data, however it runs the risk of overfitting.

##### C. Logistic regression

Logistic regression is a simple method that can be used to predict the outcome of the input variables [13]. We denote x = (x_1_, ..., x_p_) as the input variables and *y* as the response (y can be any two values). If we let *y* = 0 denote failure and *y* = 1 denote success, we have that

$$ \ln \left(\frac{\Pr \left(y=1|x\right)}{\Pr \left(y=0|x\right)}\right)={x}^T\beta +{\beta}_0 $$

or

$$ \Pr \left(y=1|x\right)=\frac{1}{1+\exp \left(-\left({x}^T\beta +{\beta}_0\right)\right)} $$

which we interpret as the probability of success (*y* = 1) given the data (*x*). The betas *β* = (*β*_1_, …, *β*_*p*_) and *β*_0_ are the coefficient values estimated from the model. Given input data, many statistical packages fit a logistic regression model and return the coefficient values. Then, given new data values *x*, we can use the above equation to estimate Pr(*y* = 1| *x*), which will be between zero and one, inclusive. Given the input *x* of an individual, we classify the individual as success if Pr(*y* = 1| *x*) > *c* where *c* is a cutoff, usually set at *c* = 0.5. Note that this method is linear in terms of parameters and will have difficulty fitting any data with a large number of input variables (*p*), even if *p* is smaller than the number of observations (*n*). In addition, if the input data follow exactly the pattern of the outcome variable, we have the so-called “complete separation” problem, which is again pronounced if we have large *p*. However, some of these shortcomings can easily be overcome with Firth’s bias adjustment [14].

##### D. Support vector machine

A support vector machine (SVM) is a machine learning method for binary classification. It uses a linear separating hyperplane *f*(*x*) = *x*^*T*^*β* + *β*_0_ to split the *p*-dimensional sample space into two groups. The SVM transforms nonlinear classification into a simpler linear classification problem, using a kernel function *K*(*x*, *x*_*i*_), with the separating hyperplane

$$ f(x)=\sum \limits_{i=1}^n{a}_i{y}_iK\left(x,{x}_i\right)+{\beta}_0 $$

and the classification criteria, sign[*f*(*x*)]. The optimization criterion is that we maximize the margin (support vector) of the separating hyperplane to obtain the optimal separation, where margin is defined as $M=\frac{1}{\mid \left|\beta \right|\mid }$, where ${\left||\beta |\right|}_2=\sqrt{\beta_1^2+\cdots +{\beta}_p^2}$. The typical choice for the kernel is a radial basis (Gaussian) kernel, *K*(*x*, *x*_*i*_) = exp(*γ*||*x* − *x*_*i*_||^2^) which is the default for most SVM software. Other kernels such as the polynomial kernel *K*(*x*, *x*_*i*_) = (1 +〈*x*, *x*_*i*_〉)^*d*^, or the neural network (hyperbolic tangent) kernel *K*(*x*, *x*_*i*_) = tanh(*κ*_1_〈*x*, *x*_*i*_〉 + *κ*_2_) may be used, but the Gaussian kernel is the most popular because it derives many desirable properties from its association with the Gaussian distribution. As the SVM method involves nonlinear kernel and optimization, it can be very computationally intensive as compared to logistic regression, but the SVM is designed for a large number of input variables (*p* > *n*), which logistic regression cannot handle.

##### E. Classification trees

The classification tree method looks for the best classification of data by splitting each variable recursively and finding the optimal combination [15]. In other words, if we are given the data y and x = (x_1_, ..., x_p_) the classification tree looks for the best split points (t_1_, ..., t_p_) that gives us the decision rule. For example, if we have three input variables *x* = (*x*_1_, *x*_2_, *x*_3_) *x*_1_, *x*_2_, *x*_3_ each taking values between 0 and 10, then the classification tree algorithm may provide the split points *t*_1_ = 5, *t*_2_, = 8, *t*_3_ = 4.5 such that we declare an input (*x*_1_, *x*_2_, *x*_3_) as success if *x*_1_ ≥ 5, *x*_2_ ≤ 8, *x*_3_ ≥ 4.5, and failure otherwise. The determination of split points largely depends on the algorithm used, for which we have many choices. Nevertheless, we see that it is easy to understand conceptually and is a popular method for classification. On the other hand, since any tree-based algorithm will involve recursively partitioning all variables to find optimal splits, generating the classification tree will also be time consuming.

##### F. AdaBoost

The classification trees, although simple to understand and implement, may be too naïve, especially if the input variables have complicated structure. The AdaBoost algorithm is a refinement of the classification tree, where the classification trees are fit recursively to determine the final classifier via a majority vote [16]. The majority voting is defined as taking the results of many sub-classifiers (here, the recursively fit trees) and making the final decision 0 or 1 from the majority of times that the sub-classifiers vote 0 or 1 [17]. The AdaBoost uses the weighted majority voting in determining the final decision, where the 0 or 1 votes are weighted based on the accuracy of sub-classifiers. Here, the weights are defined as log((err/(1-err)) at each iteration, where err is defined as weighted error rate [10]. Hence, as an iterative procedure that updates the weight at each iteration, the AdaBoost will almost always improve over classification tree. However, the AdaBoost will be even more computationally intensive than the classification trees, requiring more time to run.

##### G. Random Forest

The random forest is another method based on the classification tree [18]. Similar to AdaBoost, the random forest also involves taking a majority vote of a sample of trees to determine the final classifier, but the building of the trees involves a statistical technique known as the bootstrap. The bootstrap method repeatedly resampling the data with replacement to obtain an ensemble of trees from the original sample. To obtain the final classifier, we take a majority vote of the bootstrapped trees. Hence, the random forest is yet another recursive partitioning method that involves resampling of data. As with the AdaBoost, its performance will improve over classification tree results; however, the computation burden is greater.

#### II. Data dimension reduction methods

Classifiers, such as logistic regression, work best when the number of input variables (*p*) is smaller than the number of observations (*n*). However, if *p* > *n* or if *p* is moderately large (as is the case for our problem), we cannot correctly fit some of the classifier with the full input. Therefore, reducing the input dimension sometimes increases the performance of the classifier, and is a good practice in general for a statistical analysis. We describe here some of the dimension reduction techniques.

##### A. Principal component analysis

The most common method of reducing the dimension of input variable is the principal component analysis (PCA) [19]. In the PCA, one transforms the original variables into a set of uncorrelated orthogonal basis vectors (components), which explain monotonically decreasing amounts of variance in the original data. However, the interpretation of these bases vectors becomes difficult because the biological meaning of these principle components is not always clear, so in this paper, we adopted a technique that simply selects the most important input variables, rather than transforming them.

##### B. Shrinkage methods (ridge, LASSO, and elastic net)

Recently, the variable selection methods such as the least absolute shrinkage and selection operator (LASSO) and elastic net have gained popularity in high-dimensional statistical problems [20, 21]. We used the glmnet function from glmnet package in R, which implements the elastic net where the LASSO is a special case [22]. In the glmnet function, it is flexible to implement the ridge regression (α = 0) and LASSO (*α* = 1), and anything in between (elastic net, 0 ≤ α ≤ 1). In other words, the elastic net solves

$$ \underset{\beta_0,\beta }{\min}\left(-\left[\frac{1}{n}\sum \limits_{i=1}^n{y}_i\left({\beta}_0+{x}_i^T\beta \right)-\log \left(1+{e}^{\left({\beta}_0+{x}_i^T\beta \right)}\right)\right]+\lambda \left[\frac{\left(1-\alpha \right){\left\Vert \beta \right\Vert}_2^2}{2}+\alpha {\left\Vert \beta \right\Vert}_1\right]\right). $$

Hence, the *α* term can be flexible to control how variables are selected based on the coefficients *β*.

For our paper, we focused on LASSO (α = 1). In LASSO, the procedure simply selects variables based on the L1 hard threshold of the coefficients, ‖*β*‖_1_ (as opposed to ridge regression which only smooths out, or shrinks, the coefficients [10]). Any other *α* values smaller than one will also have the effect of shrinkage, which we do not want here. Since the (α = 1) is fixed, it remains to calculate the other parameter, *λ*, to complete the process of selecting the variables via LASSO. The glmnet function in R recommends that the users view the entire solution path consisting of results from all possible *λ* values, but practically this is unfeasible so the program selects two plausible *λ* values: *lambda.min*, the value of lambda that gives minimum mean cross-validated error, or *lambda.1se*, the largest value of lambda such that the error is within one standard-error of the minimum—the so called “one-standard-error” rule [22]. Hence, we consider two sets of selected variables based on lambda.min and lambda.1se.

Normally, one assigns test and validation sets within sample to perform classifications. However, since we have a limited sample size, we employ the *k*-fold cross validation (CV) [10]. For the *k*-fold CV, the sample is randomly divided into *k* roughly equal sized sets, and one of the *K* sets is left out. A classifier is fit with the *K* − 1 sets (training set) and validated using the remaining (left out) set. We repeat this for each of the *K* sets; the *k*-fold CV classification rate is obtained by averaging the *k* individual classification rates. Now, we repeat the process *M* times (Monte Carlo simulation of the *k*-fold CV), because the partition of the *k*-fold is random so that each time it gives us a different result. Hence, we obtain a more reliable estimate of the *k*-fold CV by repeating the procedure *M* times and also obtain the distribution of the *k*-fold CV classification rates, including the mean and the standard deviation (standard error). We set *M* = 1,000 and report the 1000 Monte Carlo mean of the *k*-fold CV classification rates. The computation times for the eight classifiers range 2–3 s for all algorithms except for AdaBoost (177 s), random forest (9 s) and KNN (< 1 s).

## Results

In this section, we present results from our simulation studies and analyses of the mitochondrial toxicity data. To obtain the classification rates, we fit the models using each of the machine learning algorithms described in the methods section and predict the outcome using simulated as well as the original mitochondrial toxicity data.

### Simulation studies

#### Simulation procedures

We ran a large set of simulation studies to assess the impact of sample size and correlation on the performance of the machine learning algorithms. We considered various sample sizes (*n*_1_ = *n*_2_ = 25, *n*_1_ = *n*_2_ = 50, *n*_1_ = *n*_2_ = 100). In each simulation, the outcome variable *D* ∈ {0, 1} (without toxicity or with toxicity) was created (for example, *n*_1_ = 25 were assigned 0 while *n*_2_ = 25 were assigned 1). For subjects with and without toxicity, we generated 12 (*p* = 12) correlated predictors from a multivariate distribution with mean vectors $\overrightarrow{1}$ and $\overrightarrow{0}$, respectively. Three distributions were considered: (1) multivariate normal, (2) multivariate-t with 3 degrees of freedom, and (3) multivariate Cauchy (t with 1 degree of freedom). We considered other distributions besides the normal distribution as some classifiers (such as LDA and SVM) perform overwhelmingly well under normality because of their algorithm structures. Due to the autoregressive nature of the mitochondrial toxicity data, we considered First Order Autoregressive *AR*(1) in addition to compound symmetry when specifying the covariance structure in our simulation. That is, for subjects with and without toxicity the predictors were generated from the aforementioned multivariate distributions with the following correlation matrices: (1) compound symmetry with off-diagonal correlation (*ρ*) values ranging from 0 to 0.9; and (2) *AR*(1) with *ρ* = 0.4 and *ρ* = 0.8. We have considered the following classifiers: Linear Discriminant Analysis (LDA), Quadratic Discriminant Analysis (QDA), K-Nearest Neighbor (KNN), Support Vector Machine (SVM), Classification Tree (CART), AdaBoost (ADA), Random Forest (RF), and Logistic Regression (LOGIT). Depending on the study, one may need to fine-tune the parameter values of the classifiers to obtain desirable results. We have tried many ways of carefully tuning the parameter values, but the tuning led to either overfitting or inaccuracy; the default values yielded a good balance between the two trade-offs and performed well in our setting. Thus, we used default parameter values with the exception of KNN (we used k = 3 instead of the default k = 1).

#### Simulation results

**Fig. 1:**
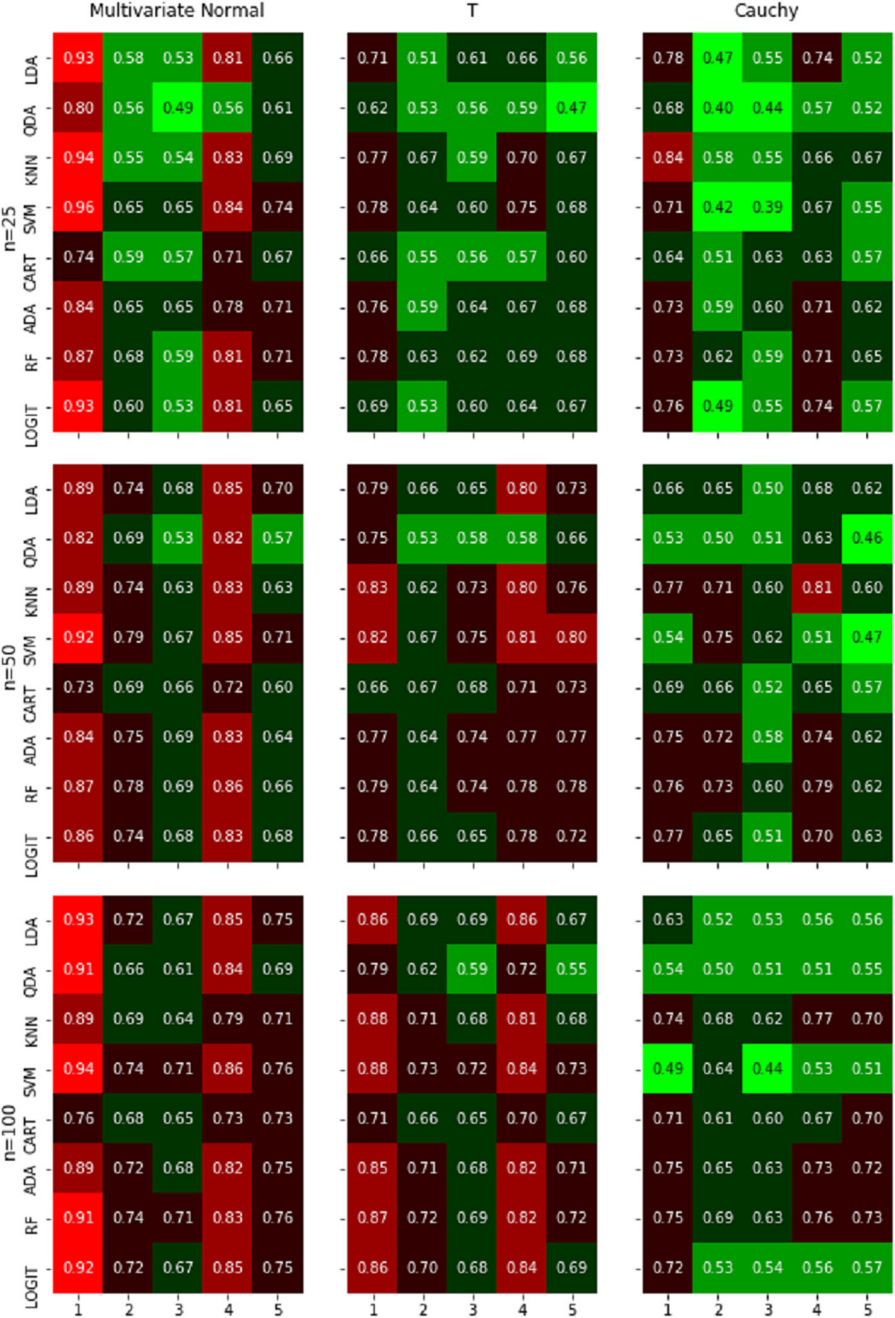
Heat map of average classification rates obtained using 5-fold CV and 1000 Monte Carlo runs. Rows represent the eight classifiers: Linear Discriminant Analysis (LDA), Quadratic Discriminant Analysis (QDA), K-Nearest Neighbor (KNN), Support Vector Machine (SVM), Classification Tree (CART), AdaBoost (ADA), Random Forest (RF), and Logistic Regression (LOGIT). Columns represent correlation matrices: compound symmetry with zero off-diagonal correlation (*ρ* = 0); compound symmetry with 0.4 off-diagonal correlation (*ρ* = 0.4); compound symmetry with 0.8 off-diagonal correlation (*ρ* = 0.8); AR(1) with *ρ* = 0.4; AR(1) with *ρ* = 0.8. Classification rate is color-coded: red, black, and green representing high, medium, and low rates, respectively

The average classification rates of our simulation studies are presented in Fig. 1 and the corresponding standard errors were all less than 0.05. The classification rates were obtained using 5-fold CV and 1000 Monte Carlo runs. As shown in the figure, the main findings of our simulation results were: (1) all the classification algorithms work better when data were least correlated, and the sample sizes were large, (2) classifiers perform better when data were normally distributed, and (3) although no single algorithm clearly out performed the others, LDA, SVM and logistic regression methods worked well under normality. KNN and tree-based methods, particularly random forest, also worked well under non-normal distributions.

We also performed simulations to assess the impact of sample size and correlation structure on data dimension reduction algorithms. More specifically, we assessed how sample size and correlation structure impacted the number of components chosen out of the 12 (*p* = 12) simulated variables. Tables 1 presents the number of components chosen (out of *p* = 12) by LASSO method. As expected, as the correlation between the 12 variables increases, the algorithm selects fewer number of variables. The *AR*(1) type correlation structure results in more selected variables compared to the compound symmetry correlation structure. The results from t and Cauchy distributions are unstable for small sample sizes thereby yielding unexpected results. Increasing in sample size resulted in an increased number of components selected.

**Table 1:** Number of components chosen by LASSO

		Sample size (*n*_1_ = *n*_2_)	*ρ* = 0	*ρ* = 0.4	*ρ* = 0.8	*AR*(1), *ρ* = 0.4	*AR*(1), *ρ* = 0.8
Normal	lambda.min	25	10	4	2	8	3
	lambda.1se	25	10	4	1	8	3
T	lambda.min	25	3	2	7	4	4
	lambda.1se	25	3	1	2	4	2
Cauchy	lambda.min	25	6	12	3	10	11
	lambda.1se	25	5	10	3	8	10
Normal	lambda.min	50	12	6	1	10	3
	lambda.1se	50	11	5	1	8	3
T	lambda.min	50	10	6	4	8	4
	lambda.1se	50	10	4	3	8	4
Cauchy	lambda.min	50	11	9	4	5	8
	lambda.1se	50	11	7	3	4	2
Normal	lambda.min	100	12	7	6	10	4
	lambda.1se	100	12	6	2	11	4
T	lambda.min	100	10	11	5	12	4
	lambda.1se	100	10	7	5	8	3
Cauchy	lambda.min	100	9	10	2	11	12
	lambda.1se	100	7	10	0	9	6

#### Data analyses

The study included 25 HIV-infected patients on NRTI-based ARTs with mitochondrial toxicity (cases), and 25 HIV-infected patients on NRTI-based ART without mitochondrial toxicity (positive controls). The median age of participants was 53 years (interquartile range (IQR), 50–57) with 60% of them being males. The race distribution among participants was 60% African American, 28% White, and 12% Hispanics. The median duration of mitochondrial toxicity was 2.2 years (IQR, 1.3–7.0). About 52% of the cases had only one manifestation of toxicity, while 48% had multiple toxicities.

A total of 12 ribose and 14 deoxyribose variables were considered in the analyses. Table 2 presents the 5-fold Monte Carlo average classification rates with corresponding standard deviations in parentheses. As shown in Table 2, the algorithms resulted in cross-validated classification rates of 0.54–0.76 for dNTP and 0.59–0.69 for RN. We have also reduced the dimension of the data using the LASSO method and applied classifiers on the reduced data. The LASSO method selects 10 deoxyribose and 2–4 ribose variables depending on whether lambda.min and lambda.1se was used as a selection criterion. For the deoxyribose data, both lambda.min and lambda.1se selects ten variables (dATP, dADP, dAMP, dTTP, dTDP, dTMP, dGTP, dGMP, dCDP, dCMP). However, for the Ribose data, lambda.min selects four variables (ATP, CDP, UTP, UMP) and lambda.1se selects two variables (ATP, UTP). Because the correlations between the deoxyribose input variables are lower than those of the ribose input variables, it is no surprise that LASSO selects most of deoxyribose input variables. Table 3 presents the 5-fold Monte Carlo average classification rates with corresponding standard deviations in parentheses for the reduced data. In general, the reduction of input variables indeed seems to improve the classification performance, particularly for the ribose dataset. The cross-validate classification rates for the ten selected dRN variables ranges from 0.65–0.76. However, cross-validate classification rates using the selected two (i.e., ATP, UTP) or four (ATP, CDP, UTP, UMP) RN variables ranged from 0.72–0.83.

**Table 2:** Average classification rates and the corresponding standard errors

	Deoxyribose	Ribose
LDA	0.69 (0.05)	0.67 (0.05)
QDA	0.64 (0.06)	0.59 (0.06)
KNN	0.54 (0.05)	0.63 (0.04)
SVM	0.58 (0.06)	0.63 (0.05)
CART	0.63 (0.06)	0.60 (0.06)
ADA	0.68 (0.04)	0.66 (0.04)
RF	0.76 (0.04)	0.69 (0.04)
LOGIT	0.67 (0.05)	0.66 (0.05)

**Table 3:** Average classification rates and the corresponding standard errors after data dimension reduction

	Deoxyribose [10]	Ribose [4]	Ribose [2]
LDA	0.73 (0.05)	0.78 (0.03)	0.74 (0.03)
QDA	0.67 (0.04)	0.73 (0.03)	0.74 (0.02)
KNN	0.65 (0.05)	0.76 (0.03)	0.76 (0.04)
SVM	0.66 (0.05)	0.80 (0.02)	0.72 (0.04)
CART	0.65 (0.06)	0.83 (0.03)	0.83 (0.02)
ADA	0.70 (0.04)	0.83 (0.02)	0.83 (0.02)
RF	0.76 (0.04)	0.77 (0.03)	0.76 (0.04)
LOGIT	0.75 (0.05)	0.76 (0.03)	0.77 (0.03)

As for which machine learning method to recommend in a classification, the tree-based methods work the best for both the deoxyribose dataset (RF) and the ribose dataset (CART and AdaBoost). But it is worthwhile noting that the simple methods such as LDA and logistic regression were very competitive in terms of classification performance. Therefore, we recommend the use of tree-based methods for this application, but if one is not comfortable with technicalities of such methods, then the simple methods such as LDA and logistic regression work as well.

## Discussion

Our study aimed to evaluate whether ART-induced mitochondrial dysfunction assessed by RN and dNTP pool sizes can be used as biomarkers in distinguishing HIV patients with mitochondrial toxicity from those without toxicity. We used eight classification algorithms to assess the diagnostic performance of RN and dNTP pool sizes distinguishing HIV patients with and without NRTI-associated mitochondrial toxicity. The algorithms resulted in cross-validated classification rates of 0.54–0.76 for dNTP and 0.59–0.69 for RN. dNTPs can be synthesized via two pathways: salvage and de novo pathways [23]. In the de novo pathway, RN is reduced to dNTP. Since there are two main sources of dNTPs, there may not be one-to-one relationship between RN and dNTP pools. This could explain the low classification rates observed in our analyses.

The reduction of input variables improved the classification performance with most of the classification algorithms. The improvement was more pronounced for RN. Due to the high correlation among the RN variables, the data reduction technique results in fewer RN variables as compared to the 10 selected dNTP variables. The cross-validate classification rates for the 10 selected dNTP variables ranges from 0.65–0.76. However, cross-validate classification rates using the selected two (i.e., ATP, UTP) or four (ATP, CDP, UTP, UMP) RN variables ranged from 0.72–0.83. The concentration of the dNTPs available during DNA replication is critical for the fidelity of DNA replication [24, 25]. dNTPs are essential precursors for DNA synthesis and perturbations in the absolute and relative concentrations of the 4 dNTPs (dATP, dTTP, dGTP and dCTP) increase mutation rates by reducing the fidelity of DNA synthesis [23]. Furthermore, imbalance in dNTP pools has been associated with mitochondrial DNA (mtDNA) mutagenesis in cell culture and animal models [26, 27]. Maintaining dNTP pools in the cell is critical not only for DNA replication but also for cell cycle control, protooncogene function, mitochondrial function, defense against viral infections, DNA mismatch repair (MMR), telomere length, mitochondrial function [24, 28, 29]. Thus, our finding of changes in RN and dNTP pools in participants with mitochondrial toxicity validates the importance of dNTP pools in mitochondrial function. Hence, RN and dNTP pools can be used as biomarkers of ART-induced mitochondrial toxicity.

In cells, the concentration of RN is several folds higher than the concentration of dNTP [30–32]. Because of the abundance of RN in the cells, its quantification is much easier than dNTP. Thus, ATP can be measured easily as biomarker of ART-induced mitochondrial dysfunction. ATP production in the cell is predominantly from mitochondria through oxidative phosphorylation (OXPHOS). Thus, ATP concentration could serve as a sensitive marker of mitochondrial function. Further studies are needed to validate our finding that ATP concentration can serve as a biomarker of ART-induced mitochondrial dysfunction. This could lead to the development of point-of-care assay for the diagnosis and monitoring of ART-induced mitochondrial toxicity.

Currently, there is no gold-standard assay for diagnosis of ART-induced mitochondrial toxicity. ART-induced mitochondrial toxicity is diagnosed by a combination of clinical symptoms, laboratory testing, and imaging studies, and sometime a tissue biopsy to demonstrate mitochondrial damage [8, 9]. Tissue biopsy is deemed to be the most accurate method. However, because it is invasive and cost prohibitive, it is seldom used in clinical practice. Moreover, not all affected organs or tissues are easily accessible for biopsy. Therefore, most providers resort to stopping the perceived offending medication to see if the clinical manifestations resolve. This practice is not optimum as it can results in the emergence of drug-resistant strains of HIV [33] and could lead to inappropriate use of second-line medications. There is a need for a non-invasive, cost-effective biomarker for ART-induced toxicity to prevent unnecessary interruptions in ART and to guide use of second-line regimens. Our finding that intracellular concentration of ATP determined by Liquid Chromatography with tandem mass spectrometry (LC/MS/MS) could be a biomarker of mitochondrial toxicity is promising. Mitochondria are responsible for ATP production through oxidative phosphorylation. Therefore, ART-induced mitochondrial dysfunction is likely to compromise ATP synthesis capacity of mitochondria. Arguments against routine use of LC/MS/MS for measuring ATP as marker of ART-induced mitochondrial toxicity are cost and labor-intensive nature of the procedure. This can be circumvented by: (1) using available and easy to use fluorometric enzyme-linked assay kits for quantifying intracellular ATP levels; or (2) using ATP concentration measured LC/MS/MS as gold standard and compare to available in vitro assays of mitochondrial function to determine which in vitro biomarkers best correlate with the level ATP measured by LC/MS/MS and, therefore, serve as the “go-to” assay(s) for diagnosing and monitoring ART-induced mitochondrial toxicity.

## Conclusions

We used a range of machine learning procedures to distinguish between HIV patients with and without toxicity. Using resampling methods like Monte Carlo *k*-fold cross validation, we compared the accuracy of several machine learning algorithms applied to our data. We used the algorithm with highest classification accuracy rate in evaluating the diagnostic performance of RN and dNTP pool sizes as biomarkers of mitochondrial toxicity. The algorithms resulted in cross-validated classification rates of 0.65–0.76 for dNTP and 0.72–0.83 for RN. Our finding of changes in RN and dNTP pools in participants with mitochondrial toxicity validates the importance of dNTP pools in mitochondrial function. Hence, levels of RN and dNTP pools can be used as biomarkers of ART-induced mitochondrial toxicity. There are none invasive and cost-effective assays to measure intracellular ATP concentration that could be used to monitor or diagnose ART-induced mitochondrial toxicity.

## Data Availability

The dataset used in the manuscript is available from the corresponding author on reasonable request.

## Abbreviations

ADA: AdaBoost
AIDS: Acquired immunodeficiency syndrome
AR (1): First order autoregressive
ART: Antiretroviral therapy
CART: Classification and regression tree
CV: Cross validation
DNA: Deoxyribonucleic acid
dNTPs: deoxyribonucleotide triphosphates
dNTTP: deoxyribonucleotide
HIV: Human immunodeficiency virus
IQR: Interquartile range
KNN: K-nearest neighbor
LASSO: Least absolute shrinkage and selection operator
LDA: Linear discriminant analysis
LOGIT: Logistic regression
mtDNA: mitochondrial DNA
NRTIs: Nucleoside reverse transcriptase inhibitors
PCA: Principal component analysis
PIs: protease inhibitors
QDA: Quadratic discriminant analysis
RF: Random forest
RN: Ribonucleotides
SVM: Support vector machine
